# Modeling induced resistance to plant disease using a dynamical systems approach

**DOI:** 10.3389/fpls.2013.00019

**Published:** 2013-02-18

**Authors:** Nurul S. Abdul Latif, Graeme C. Wake, Tony Reglinski, Philip A. G. Elmer, Joseph T. Taylor

**Affiliations:** ^1^Faculty of Agro Based Industry, Universiti Malaysia KelantanJeli, Kelantan, Malaysia; ^2^Institute of Information and Mathematical Sciences, Massey UniversityAuckland, New Zealand; ^3^The New Zealand Institute for Plant and Food Research LimitedHamilton, New Zealand

Standard epidemiological models describe that **Susceptible** plants (*S*) will become infected and develop **Disease** (*D*) after inoculation with a compatible pathogen under appropriate environmental conditions. These dynamic relationships can be affected by subtle changes to any one parameter and may result in a proportion of the plant population being able to exhibit **Resistance** (*R*) to infection. An example of this is the use of elicitors to promote an increase in plant basal resistance and so enable a proportion of formerly susceptible plants to express resistance i.e., a shift in the population from *S* to *R*. This phenomenon is termed induced resistance (IR). In this paper, a prototype mathematical model of IR is presented to describe the effects of a chemical elicitor compound, methyl jasmonate (MeJA), on the resistance of *Pinus radiata* seedlings to *Diplodia pinea* the causal agent of pine stem canker and tip dieback. Pine seedlings were sprayed with 0.1% MeJA at 27, 13, 6, or 3 days before inoculation with *D. pinea* using methods previously described by Gould et al. (2008). Disease assessments commenced at 1 week after inoculation and continued at 3–4 day intervals thereafter for 5 weeks. Disease development on the MeJA-treated seedlings was compared to that on a cohort of untreated plants. In this model system, the IR response is transient and it is modeled here using a forward-and-backward kinetics framework to describe the temporal nature of the phenomenon.

Because the expression of IR can only be detected after pathogen challenge, the model is formulated with the treated plants divided into two regimes: (1) pre-inoculation and (2) post-inoculation. The assumptions for the model's formulation can be summarized as follows. The plant population is divided into three compartments according to the above definitions where *S* + *R* + *D* = 1. At the time when plants are treated with an elicitor (*t* = 0), a proportion of the plant population will exhibit natural resistance (*R*_*i*_). The induction period (*t*_*p*_) describes the time interval between elicitor application and pathogen inoculation. Upon inoculation, a proportion of plants (*D*_*i*_) will become infected immediately. This prototype IR model is based on the model by Jeger et al. (2009) and Xu et al. (2010). The model's equations for the **treated** plants are as follows:

Pre-inoculation: For 0 ≤ *t* < *t*_*p*_

(1) $$\begin{array}{l} \;\frac{dR}{dt}=(e(t)-\gamma R)(1-R);\\ R(0)=R_{i} \end{array}$$

Post-inoculation: For *t*_*p*_ ≤ *t* ≤ *T*

(2) $$\begin{array}{l} \;\frac{dR}{dt}=(e(t)-\gamma R)(1-R-D);\\ R(t_{p})=R_{p} \end{array}$$

(3) $$\begin{array}{l} \;\frac{dD}{dt}=\beta D(1-R-D);\\ D(t_{p})=D_{i} \end{array}$$

where we take $e(t)=\frac{kt}{t^{2}+L^{2}}$ [days^−1^] as the elicitor effectiveness in the plants where $\frac{k}{2L}$ [days^−1^] is the maximum elicitor effect and *L* [days] is the time where this is at its peak, γ [days^−1^] is the rate that resistant tissue becomes susceptible, and also β [days^−1^] is the rate of disease development. The form for *e*(*t*) is chosen to reflect the temporal nature of IR with an initial increase in resistance which then decays over time. *R*_*p*_ is the degree of the resistance at the time of the pathogen inoculation obtained from Equation (1). Also, *T* is a finite (sufficiently large) time after the pathogen inoculation. The Equations (1)–(3) are based on the assumption that the rate of change of *R* and *D* are directly proportional to the amount of *S* available at a particular time (that is, *S* = 1 − *R* − *D*). For Equation (1), *D* is not in the equation because the pathogen is absent during this period. Pathogen is inoculated at time *t*_*p*_, therefore the *D* term is occurs only in Equations (2) and (3). For the untreated plants, they share the same parameter values, especially β, *R*_*i*_, *D*_*i*_, since these untreated plants are characterized as the control group. The untreated plants will not have the *e*(*t*) term in their model equations and so are autonomous, that is, independent of time except implicitly. Therefore, the model's equations for the **untreated** plants are as follows:

(4) $$\begin{array}{l} \frac{dR}{dt}=\alpha R(1-R-D);\\ R(0)=R_{i} \end{array}$$

(5) $$\begin{array}{l} \frac{dD}{dt}=\beta D(1-R-D);\\ D(0)=D_{i} \end{array}$$

where α [days^−1^] is the rate that untreated susceptible tissue becomes resistant.

The seven unknown parameters (α, β, γ, *k*, *L*, *R*_*i*_, *D*_*i*_) are determined by matching data to the model using computer-based algorithms. The following figure (Figure 1) was plotted using the optimal parameter values, and it illustrates the characteristics of the two compartments *R* and *D* for the Equations (1)–(5). The flows in the figure indicate the time-evolution of the system of the differential equations described above based on the different values of *t*_*p*_ and the untreated case. It shows that when *t*_*p*_ is between 3 and 6 days, the subsequent development of disease is less severe than with the other induction times and with the untreated plants. That is, the resistance induced by the elicitor application is at its peak. The figure shows that the trajectories for each induction case will eventually approach that straight line *R* + *D* = 1. The model has three compartments (*S*, *R*, *D*), but the data sets have only two ([*S* + *R*], *D*). The Susceptible cohort is given by *S* = 1 − *R* − *D* and can be separated out by this model. The model has a line of equilibrium states in the *R*, *D* plane which are attracting, and predicts that disease development depends on the induction time *t*_*p*_.

**Figure 1:**
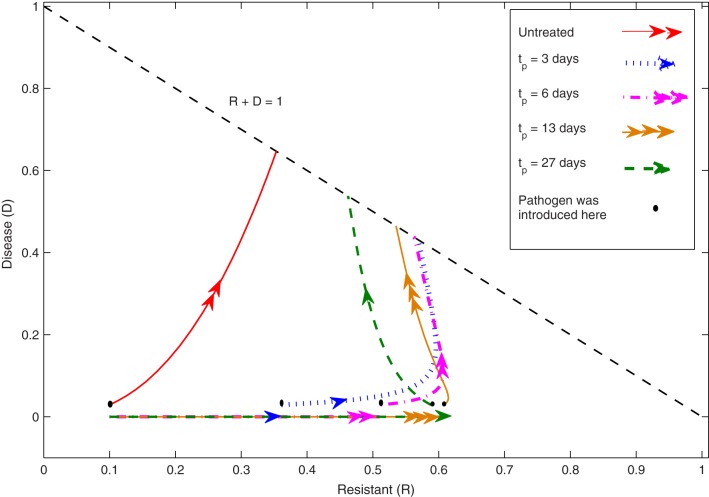
**The phase-plane for the induced resistance (IR) model.** These trajectories of the *R*, *D* compartments are plotted in the feasible region (*R*, *D* > 0, *R* + *D* < 1). The lines schematically represent the values of the two compartments *R* and *D* as time passes for the Equations (1)–(5) based on each induction case *t*_*p*_ and the untreated case. When *D* = 0, the straight lines illustrate the dynamics of the *R* compartment before the pathogen inoculation. These lines will have a discontinuity when the pathogen is introduced at time *t* = *t*_*p*_, and it shows the state of the *R* compartment at that particular time. As can be seen in the figure, there is a jump in the *D* values of *D_0_* at *t* = *t*_*p*_ and the trajectories of the *R*, *D* compartments will continue to approach the straight line *R* + *D* = 1. For the untreated case, the dynamics of the *R*, *D* compartments will depend on the initial condition of the systems i.e., *R*_*i*_ and *D*_*i*_.

## Conclusion

The management of plant diseases involves an assessment of the risks and the costs, both economic and environmental, associated with the implementation of different control measures. Various disease risk prediction models have been developed as decision support tools to facilitate more efficient use of management options; these are generally based on the rationale that pest and disease development follow predictable life cycles and that by monitoring key epidemiological parameters it is possible to target more accurately events critical for management (Gent et al., 2011). Disease risk prediction models may prove critical for coordination of elicitor application in crop production systems because of the importance of early intervention when relying on IR for disease control. In this study we discuss the development of a prototype mathematical model to predict the temporal dynamics of chemically-IR. The current model offers the potential to quantitatively estimate the effectiveness of elicitor treatment and to predict the relative proportion of plants exhibiting IR to pathogen inoculation. Moreover, the model is generic and will be applicable for a range of plant-pathogen-elicitor scenarios. For future work, this prototype IR model will be extended to predict the required *t*_*p*_ to achieve optimum disease control. In addition, it will be interesting to observe the dynamics of the system when there are multiple elicitor applications to plants, a scenario which may be needed in practice. This is important in practical terms because successful application of elicitors will require knowledge of the onset and duration of the IR response. This new model will complement and extend the value of risk prediction models by providing decision support on the timing and frequency of elicitor applications for management of disease.
